# Effects of Uremic Clearance Granules in Uremic Pruritus: A Meta-Analysis

**DOI:** 10.3390/toxins13100702

**Published:** 2021-10-04

**Authors:** Ping-Hsun Lu, Jen-Yu Wang, Hui-En Chuo, Po-Hsuan Lu

**Affiliations:** 1 Department of Chinese Medicine, Taipei Tzu Chi Hospital, Buddhist Tzu Chi Medical Foundation, New Taipei City 23142, Taiwan; 101318121@gms.tcu.edu.tw; 2 School of Post-Baccalaureate Chinese Medicine, Tzu Chi University, Hualien 97048, Taiwan; 3 MacKay Junior College of Medicine, Nursing, and Management, New Taipei City 11260, Taiwan; jyw.6993@mmh.org.tw; 4 Department of Dermatology, MacKay Memorial Hospital, Taipei 10449, Taiwan; waynec.4204@mmh.org.tw; 5 Department of Medicine, MacKay Medical College, New Taipei City 252005, Taiwan

**Keywords:** uremic clearance granules, chronic kidney disease, uremic pruritus, inflammatory biomarkers, mineral metabolism

## Abstract

Uremic pruritus is common among patients with advanced or end-stage renal disease, with an incidence of >40% among patients on dialysis. Uremic clearance granules (UCGs) are effective in managing uremic pruritus and delay the progression of chronic kidney disease. We conducted a systematic review and a meta-analysis to evaluate the efficacy of UCG in patients with uremic pruritus. Several electronic databases were searched systematically from their inceptions until 19 July 2021. Randomized control trials evaluating the efficacy of UCG in patients with uremic pruritus were selected. Eleven trials including 894 participants were published between 2011 and 2021. Patients administered UCGs had a significantly decreased visual analog scale score (mean difference [MD], −2.02; 95% confidence interval [CI], −2.17 to −1.88), serum levels of hsCRP (MD, −2.07 mg/dL; 95% CI, −2.89 to −1.25; *p* < 0.00001), TNF-α (MD, −15.23 mg/L; 95% CI, −20.00 to −10.47; *p* < 0.00001]), β2-MG (MD, −10.18 mg/L; 95% CI, −15.43 to −4.93; *p* < 0.00001), and IL-6 (MD, −6.13 mg/L; 95% CI, −7.42 to −4.84; *p* < 0.00001). In addition, UCGs significantly reduced serum levels of creatinine, BUN, PTH, iPTH, phosphorus, and the overall effectiveness rate. UCGs could be an attractive complementary therapy for patients with uremic pruritus.

## 1. Introduction

Uremic pruritus (UP) is a distressing complication in patients with end-stage renal disease (ESRD) and on dialysis, either hemodialysis (HD) or peritoneal dialysis (PD) [1]. Aside from generalized pruritus, UP causes sleep disturbance, depression, poor quality of life, and increased mortality risk [2,3]. Several markers have been reported to be associated with UP. Conventionally, dialysis efficiency and mineral metabolism markers, for example, phosphorus (P), calcium (Ca), parathyroid hormone (PTH), and intact parathyroid hormone (iPTH) levels, were thought to be associated with increased risk of UP [4,5,6]. Recently, UP has been considered a systemic inflammatory disease. The association of inflammatory markers, such as C-reactive protein (CRP), interleukin-6 (IL-6), interleukin-2 (IL-2), β2-microglobulin (β2-MG), and tumor necrosis factor-α (TNF-α) with UP has been confirmed [7,8,9]. Therapies for UP include systemic treatments, topical treatment, and complementary alternative medicine [3,10,11]. Furthermore, only a few meta-analyses on the use of treatment agents such as gabapentin and nalfurafine with some adverse effects exist [10,11]. Although the use of acupressure has also been reported, the evidence is insufficient [12]. Since Chinese herbal bath therapy depletes body fluids, it is unsuitable for weak patients [13]. Hence, identifying complementary therapies for UP with fewer adverse effects is critical.

Studies have reported the benefits of oral and topical application of traditional Chinese medicine (TCM) in UP [13,14]. Uremic clearance granules (UCGs) (Consun Pharmaceutical Group), or NiaoDuQing, were developed based on TCM principles and have been widely used for >20 years to treat chronic kidney disease (CKD) [15]. UCGs are the first TCM agents to be approved for CKD treatment by the China Food and Drug Administration (National Medicine Permit No. Z20073356) [16]. According to the TCM theory, UP is a yin deficiency of the kidney and spleen, which causes dampness and toxin accumulation. Furthermore, yin deficiency produces heat and blood deficiency produces wind. The UP syndrome, according to TCM, involves dampness, toxins, wind, fire, and stasis, which interact with each other during UP [17,18]. The prescription for UCG in TCM includes 16 herbs, such as Danshen, Gancao, Huangqi, Fuling, Kushen, Juhua, and so forth (Table 1) [19,20]. These herbs contain various biologically active compounds such as salvianolic acid A, paeoniflorin, emodin, isoflavone, and astragaloside IV [21]. These compounds show synergistic effects to improve renal function, including lowering blood urea nitrogen (BUN) and serum creatinine (Scr) levels [15]. balancing the Ca and P metabolic dysfunction [22]. improving the systemic microinflammatory state [23], and reducing the severity of UP [24]. In this study, we performed a systematic literature review and meta-analysis of eligible randomized controlled trials (RCTs) to evaluate the efficacy of UCG in the management of UP.

## 2. Results

### 2.1. Study Characteristics

**Figure 1:**
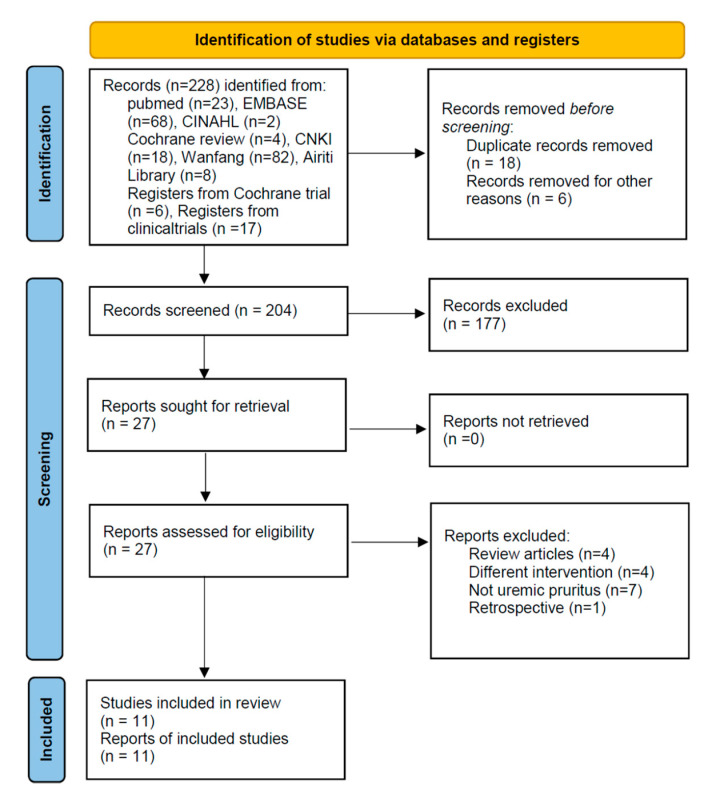
PRISMA 2020 Flow Diagram.

The described search strategy yielded 228 articles (five English, three Chinese; 24 duplicate studies were excluded). After screening the title and abstract, we excluded 177 articles per our study selection criteria earlier. Full texts of the remaining 27 articles were reviewed; 16 articles were excluded for the following reasons: four were review articles [25,26,27,28], four applied a different intervention [29,30,31,32], seven did not include patients with UP [15,33,34,35,36,37,38], and one was a retrospective study [39]. Finally, 11 RCTs were included in the meta-analysis (Figure 1) [24,40,41,42,43,44,45,46,47,48,49].

The basic characteristics of the 11 RCTs published between 2012 and 2021 are listed in Table 2 and Table S1. A total of 894 participants were enrolled, with the sample size ranging from 32 to 128. All included studies were RCTs that recruited patients with UP experiencing itching and on maintenance HD; most participants were adults. The patients received HD combined with hemoperfusion (HP) in two trials [45,47] and HD combined with Caltrate D600 and Calcitriol in one [41]. All these trials were included in our meta-analysis as they met the inclusion criteria.

**Figure 2:**
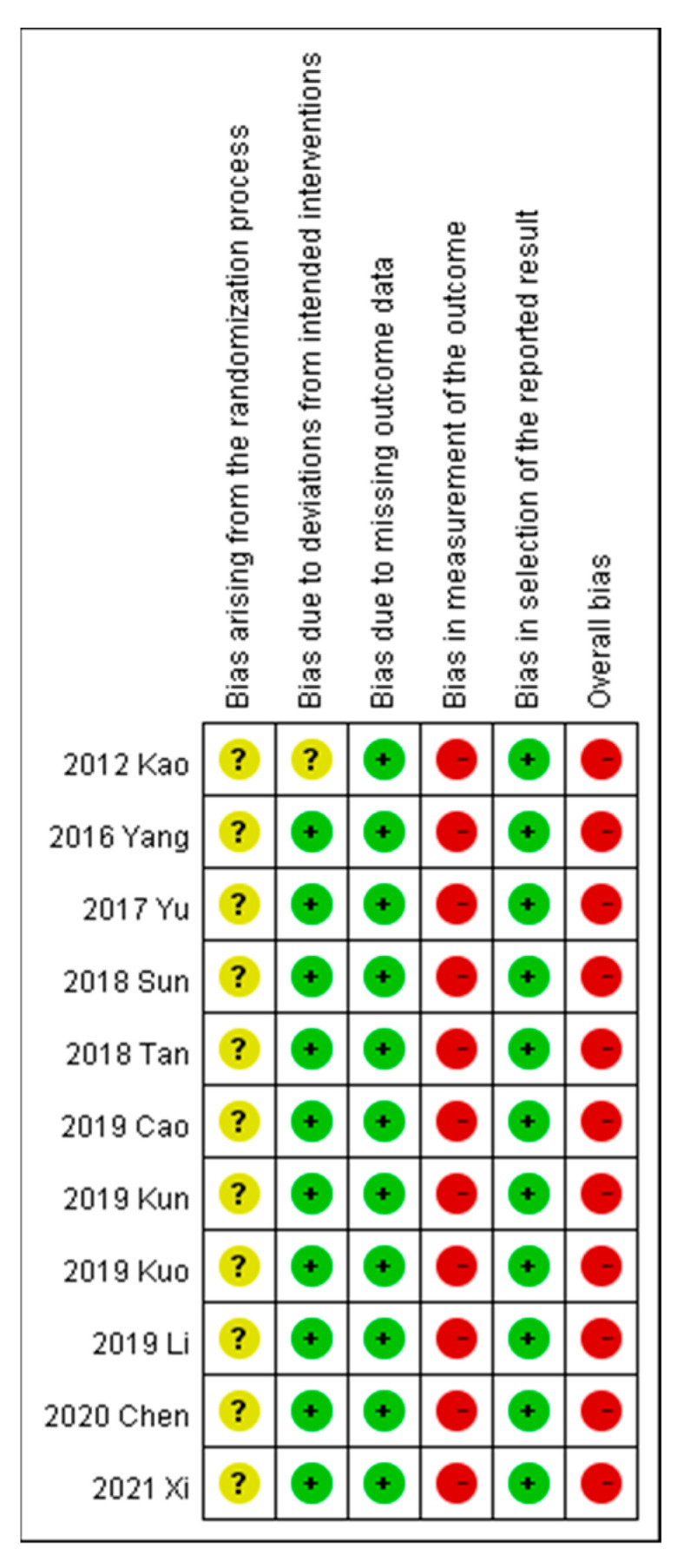
Risk of bias in different studies.

Risk of bias was assessed per the recommendations of RoB 2 (revised tool to assess the risk of bias in randomized trials; latest revision, 22 August 2019), as shown in Figure 2. Random allocation was applied in all included trials, but none offered information about the concealed allocation sequences. With regard to randomization, three trials followed the visit order [40,45,49], seven studies used a randomization table [24,42,43,44,46,47,48], and one study used random sampling [40]. One study provided no information on baseline characteristics [47]. Double blinding was not used in any trial, and an intention-to-treat analysis with an acceptable loss-to-follow-up rate (< 5%) was used. Approximately all the outcome data were available in the included trials. The outcome measurement bias for the primary outcomes, visual analog scale (VAS), and overall effectiveness rate, was high because these were reported by the participants. The outcome measurement bias for the secondary outcomes was low as these laboratory data were not affected by patient blinding.

### 2.2. Primary Outcomes

**Figure 3:**
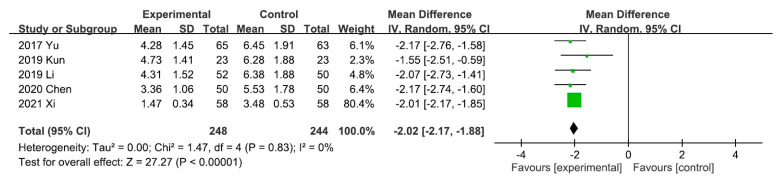
Forest plot for comparison of the visual analog scale in patients with uremic pruritus treated with uremic clearance granules. CI, confidence interval.

**Figure 4:**
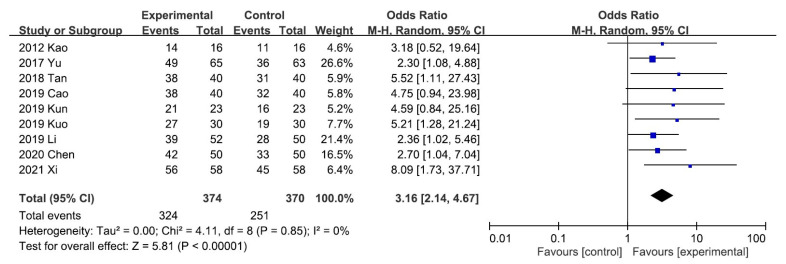
Forest plot for comparison of the overall effectiveness in patients with uremic pruritus treated with uremic clearance granules. CI, confidence interval.

A meta-analysis of the five trials scoring pruritus using the VAS showed that the VAS score decreased significantly after UCG administration (mean difference [MD], −2.02; 95% CI, −2.17 to −1.88]. Heterogeneity across the trials was not significant (*I^2^* = 0%; *p* < 0.00001; Figure 3). A meta-analysis of the night trials using itch scales (Dirk R. Kuypers, Duo, and Mettang, or 5-D itch scale) showed that the overall effectiveness in the UCG group was significantly higher than that in the placebo group (odds ratio [OR], 3.16; 95% CI, 2.14–4.67). Heterogeneity across trials was not significant (*I^2^* = 0%; *p* < 0.00001; Figure 4).

### 2.3. Secondary Outcomes

#### 2.3.1. Effect of UCG on Serum Levels of Ca, P, PTH, and iPTH

**Figure 5:**
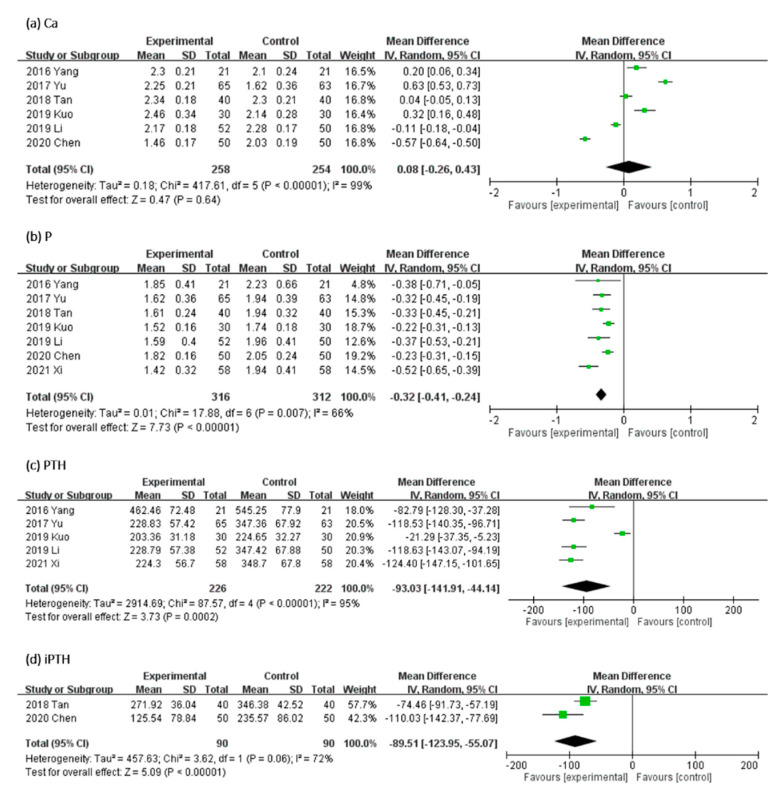
Forest plot for comparison of the serum level of (**a**) Calcium (Ca), (**b**) Phosphorus (P), (**c**) parathyroid hormone (PTH), and (**d**) intact parathyroid hormone (iPTH) in patients with uremic pruritus treated with uremic clearance granules. CI, confidence interval.

In Figure 5, the effect of UCG supplementation on Ca, P, PTH, and iPTH serum levels was assessed as follows: serum level of Ca in six, P in seven, PTH in five, and iPTH in two trials. Results of our meta-analyses revealed that UCG supplementation significantly decreased the serum P level (MD, −0.32 mmol/L; 95% CI, −0.41 to −0.24; *p* < 0.00001), PTH (MD, −93.03 ng/L; 95% CI, −141.91 to −44.14; *p* = 0.0002), and iPTH (MD, −89.51 mmol/L; 95% CI, −123.95 to −55.07; *p* < 0.00001), but not Ca (MD, 0.08 mmol/L; 95% CI, −0.26 to −0.43; *p* = 0.64). Heterogeneity across trials was high for Ca, PTH, iPTH, and P (Ca: *I^2^* = 99%; *p* < 0.00001; PTH: *I^2^* = 95%; *p* < 0.00001; iPTH: *I^2^* = 72%; *p* = 0.06; P: *I^2^* = 66%; *p* = 0.007). 

#### 2.3.2. Effect of UCG on SCr, BUN, and Uric Acid Levels

**Figure 6:**
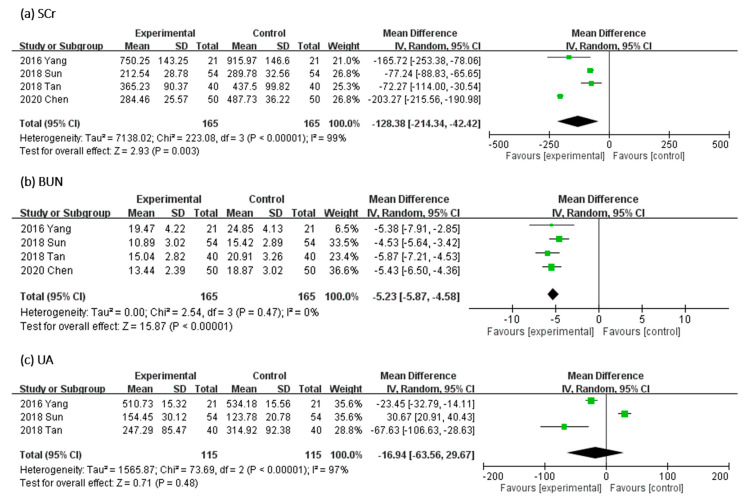
Forest plot for comparison of the serum level of (**a**) serum creatinine (SCr), (**b**) blood urea nitrogen (BUN), and (**c**) uric acid (UA) in patients with uremic pruritus treated with uremic clearance granules. CI, confidence interval.

In Figure 6, the effect of UCG supplementation on SCr and BUN was assessed in four trials and of uric acid (UA) in three RCTs. Results of our meta-analyses revealed that UCG supplementation significantly decreased the Scr level (MD, −128.38 μmol/L; 95% CI, −214.34 to −42.42; *p* = 0.003) and BUN level (MD, −5.23 mmol/L; 95% CI, −5.87 to −4.58; *p* < 0.00001) but not the UA level (MD, −16.94 μmol/L; 95% CI, −63.56 to 29.67; *p* = 0.48). The heterogeneity across trials was high for Scr (*I^2^* = 99%; *p* < 0.00001) and UA (*I^2^* = 97%; *p* < 0.00001); however, heterogeneity for BUN was not significant (*I^2^* = 0%; *p* = 0.47).

#### 2.3.3. Effect of UCG on Serum Levels of Inflammatory Biomarkers

**Figure 7:**
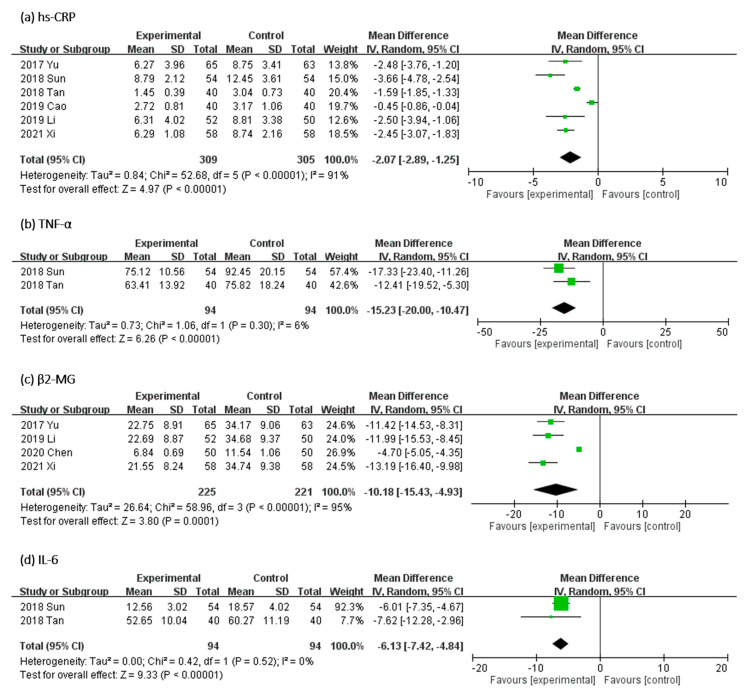
Forest plot for comparison of the serum level of inflammation biomarkers (**a**) high-sensitivity C-reactive protein (hs-CRP), (**b**) tumor necrosis factor-α (TNF-α), (**c**) β2-microglobulin (β2-M), and (**d**) interleukin-6 (IL-6) in patients with uremic pruritus treated with uremic clearance granules. CI, confidence interval.

In Figure 7, the effect of UCG supplementation on the serum levels of inflammatory biomarkers was evaluated as follows: high-sensitivity C-reactive protein (hs-CRP) in six RCTs, β2-MG in four RCTs, TNF-α and IL-6 in two trials. Results of our meta-analyses revealed that UCG supplementation significantly reduced the serum levels of hs-CRP (MD, −2.07 mg/dL; 95% CI, −2.89 to −1.25; *p* < 0.00001), β2-MG (MD, −10.18 mg/L; 95% CI, −15.43 to −4.93; *p* < 0.00001), TNF-α (MD, −15.23 mg/L; 95% CI, −20.00 to −10.47; *p* < 0.00001), and IL-6 (MD, −6.13 mg/L; 95% CI, −7.42 to −4.84; *p* < 0.00001). Heterogeneity across trials was high for hs-CRP (*I^2^* = 91%; *p* < 0.00001) and β2-MG (*I^2^* = 95%; *p* < 0.00001), but low for TNF-α (*I^2^* = 6%; *p* = 0.30) and IL-6 (*I^2^* = 0%; *p* = 0.52).

### 2.4. Quality of Evidence

Given the high risk of bias for the primary outcomes, the quality of evidence was low for UCG administration in UP patients (Table S2).

## 3. Discussion

Our meta-analysis suggests that UCG administration significantly reduces the VAS score and improves the overall effectiveness rate. UCG supplementation also significantly decreases the BUN, Scr, PTH, iPTH, and serum P levels and reduces the inflammatory biomarkers. However, further studies with larger sample sizes and rigorous designs focusing on different dosages and routes of UCG supplementation in patients on both HD and PD are recommended. Our meta-analysis revealed that UCG administration significantly reduced the VAS score and improved the overall effectiveness rate. The serum levels of P, PTH, iPTH, Scr, and BUN decreased significantly. Only one of ten studies in our meta-analysis reported an adverse effect, although no statistical significance was seen in comparison with the control group. UCG supplementation improves outcomes with regard to inflammatory biomarkers, P, PTH, iPTH, Scr, and BUN levels and reduces the itching sensation.

Systemic inflammation caused by pruritogens (accumulated uremic toxins, histamines, and proinflammatory cytokines) and the imbalance of the opioid system are considered to be possible pathogenesis of UP [5,50]. The accumulated uremic toxins can destroy the intestinal barrier integrity, cause dysbiosis of the gut, and progressive kidney damage [50,51]. Toxins, including mineral products and uremic toxins, were proposed as pruritogens [4]. Previous studies have also proposed that UP is associated with abnormal mineral metabolism (e.g., higher serum Ca, P, ferritin, and PTH levels) [3,6,52,53]; however, recent studies reported conflicting results [4]. The study by Rayner et al. showed that older age, higher CRP level, low serum albumin level, and presence of hepatitis B or C were associated with a higher risk of developing an itchy skin, and UP was not associated with serum P, Ca, PTH, Kt/V, and hemodiafiltration [54]. Ozen et al. also reported no association between UP and mineral metabolism [55]. In addition, β2-MG has been considered an inflammatory factor [56] and a pruritogen, eliciting itch-related responses [57]. One study found that patients with uremia had elevated IL-6, TNF-α, and CRP levels [58]. Kimmel et al. reported that serum CRP, IL6, and TNF-α levels were higher in patients on HD with UP than in those without UP [9]. The serum hs-CRP level and the severity of pruritus are lower among patients with UP who are vegetarian or switched to a vegetarian diet [59]. Studies have also shown that administering omega-3 to patients on HD reduces serum hs-CRP levels [60] and pruritus scores [61]. The aforementioned findings suggest that UP is associated with microinflammation in the skin or systemic inflammation [4]. In our meta-analysis, administration of UCG to patients with UP significantly decreased the metabolic biomarker levels (e.g., Scr, BUN, P, and PTH) and inflammatory markers (e.g., hs-CRP, TNF-α, β2-MG, and IL-6). The importance of inflammation and proinflammatory factors in patients with UP needs further investigation.

In addition, oral activated charcoal and hemoperfusion could lower uremic toxins and change the microbiota [50]. TCM also showed the ability to reduce different kinds of uremic toxins and modulate intestinal microbiota [51]. UCG can reduce Scr levels [15], resolve Ca and P metabolic dysfunction [22], and improve systemic microinflammation [23] to delay CKD progression. A meta-analysis revealed that in patients with CKD III-V receiving UCG, the Scr, triglyceride, cholesterol levels could be reduced and the hemoglobin level and glomerular filtration rate could be increased [62]. Administration of UCG decreased the serum protein-bound uremic toxins such as indoxyl sulfate and p-cresyl sulfate in patients with ESRD by modifying intestinal microbiota or reducing kidney oxidative stress and inflammation [63,64]. Rhubarb, an ingredient in UCG, showed the benefit of gut microbiota modulation and inflammation alleviation enhancing the amount of probiotic *Lactobacillus* and short-chain fatty acid-producing bacteria [65].

In the rat renal failure model, UCG showed a renoprotective effect; it could promote extracellular matrix degradation and regulate MMP-2/TIMP-1 balance or signal molecular activity of the TGF-β/Smad pathway to alleviate renal dysfunction and tubulointerstitial fibrosis [66]. UCG could decrease renal fibrosis and anemia in CKD rats by regulating the transforming growth factor-β (TGF-β) and erythropoietin signaling pathways. These pharmacologic results were consistent with in silico predictions [16].

Among the RCTs included in our study, UCG dosage varied from 5 to 20 g/day for oral and 30 g/day for enema administration; moreover, the treatment duration also varied from one month to three months for oral and 18 months for enema. The overall effectiveness of oral and enema UCG was similar in our study (75.0%–95.0% vs. 87.5%) [24,40,41,42,43,45,48,49]. In another RCT on UP therapy, the enema UCG group showed a higher overall effectiveness rate than the oral UCG group (74.19% vs. 41.94%) [67]. Although RCTs have demonstrated the beneficial effects of UCG supplementation, the optimal dosage, route, and duration of administration remain unclear. The study by Tan used UCG along with HD and HP and showed a better antipruritic effect than the control group receiving only HD and HP [45].

The studies included in our meta-analysis showed considerable heterogeneity because of various clinical factors. First, the route, dosage, and duration of UCG differed across the studies. Second, some discrepancies in the interventions of the control groups existed. Most of the trials used HD [24,40,42,43,44,48,49], two mn trials used HD and HP [45,47], and another trial used HD, oral Caltrate D600, and Calcitriol [41]. Finally, outcomes of overall effectiveness rate were measured using different pruritus scales. These inconsistencies between trials resulted in heterogeneity. No significant heterogeneity was seen across trials in VAS, overall effectiveness rate, P, BUN, TNF-α, and IL-6. This study has some limitations. First, the included RCTs had small sample sizes. The method of randomization was unclear, and no trial reported double blinding. For pruritus evaluation, more detailed and multidimensional pruritus assessments considering not only pruritus severity and direction but also life quality such as 5D-IS and 12-item pruritus severity scale are needed [68,69,70]. Uniform pruritus scale is better for comparison of therapeutic effect.

## 4. Conclusions

Our meta-analysis suggests that UCG administration significantly reduces the VAS score and improves the overall effectiveness rate. UCG supplementation also significantly decreases the BUN, Scr, PTH, iPTH, and serum P levels and reduces the inflammatory biomarkers. However, further studies with larger sample sizes and rigorous designs focusing on different dosages and routes of UCG supplementation in patients on both HD and PD are recommended.

## 5. Materials and Methods

### 5.1. Literature Search

Articles published until 19 July 2021 were searched for on the PubMed, Embase, Cochrane Library, CINAHL, ClinicalTrials.gov, Chinese National Knowledge Infrastructure, Airiti Library, and Wanfang databases. The MeSH and Emtree search headings were used as follows: uremic clearance granule, pruritus, uremic, chronic kidney disease, dialysis, and their synonyms. We conducted free-text search using these terms and their combinations. To broaden the search, we also reviewed all the retrieved articles and citations using the “related articles” facility on PubMed. Furthermore, we parsed the cited studies in the accessed papers manually and contacted known experts of the field to identify other studies. Finally, unpublished studies were inspected from the ClinicalTrials.gov registry (http://clinicaltrials.gov/ (accessed on 19 July 2021)). The search was not restricted by language. This systematic review was accepted by the online PROSPERO of the National Institute for Health Research (CRD 42020199834). The complete search strategy is described in the supplement (Table S3).

### 5.2. Study Selection

RCTs were selected to evaluate the efficacy of UCG in UP patients. The inclusion criteria were as follows: the presence of UP, administration of UCG, and availability of quantitative data for pruritus severity. We excluded studies wherein UP was not diagnosed. We included studies regardless of the pruritus evaluation methods. When raw or missing data were required, we contacted the authors by email. We selected the study with the larger population when duplicate studies published using overlapping data sets.

### 5.3. Data Extraction and Quality Assessment

Two authors extracted the following information from each article independently: publication details, study design, inclusion and exclusion criteria, matching criteria, sample size, age, intervention period, route, dosage and frequency of UCG and placebo, pruritus severity assessment tool, quantification of pruritus severity, renal function, inflammation biomarkers, serum levels of Ca, P, PTH, and iPTH, and the overall effectiveness rate. The selected studies were screened for eligibility by two reviewers; subsequently, the aforementioned inclusion criteria were applied. The reviewers reviewed the articles independently and compared the results; discrepancy was resolved by a third reviewer. The quality of the studies was evaluated using the “risk of bias” method recommended by the Cochrane Collaboration [71].

### 5.4. Data Synthesis and Analysis

The efficacy of UCGs was evaluated using outcome measures described herein. The primary outcomes included mean differences in VAS scores and the OR of the overall effectiveness rate. Secondary outcomes included the mean differences in serum Ca, P, PTH, and iPTH levels; renal function indexes; and inflammation biomarkers. We conducted the analysis using RevMan 5.4 (Cochrane Collaboration, Copenhagen, Denmark). A meta-analysis was conducted according to recommendations in the PRISMA guidelines. We used the OR as the summary statistic to analyze the dichotomous outcomes and used the weighted mean difference (WMD) to analyze the continuous outcomes. Both the summary statistics were reported with 95% confidence intervals (CIs). The random-effect model was used to pool OR and WMD estimates if studies were statistically heterogeneous. The *I^2^* test and Cochran Q statistic were used to assess heterogeneity among studies. The pooled estimates were considered to show significant heterogeneity when *I^2^* > 50% or *p* < 0.1. The quality of evidence was assessed using the Guideline Development Tool developed by the GRADE Working Group [72].

## Figures and Tables

**Table 1:** Overview ingredients of UCG.

Chinese Name	English/Latin Name	Family	Species	Prescription Functions (TCM Patterns)
Dahuang	Rheum palmatum/*Rheum officinale*	Polygonaceae	*R. officinale*	To drain heat, cool blood, resolve toxins, and expel stasis
Gancao	Glycyrrhiza inflata/*Glycyrrhiza uralensis*	Fabaceae	*G. uralensis*	To supplement center and boost qi, drain fire, and resolve toxins
Chaihu	Bupleurum scorzonerifolium/*Bupleurum chinense*	Apiaceae	-	To harmonize exterior and interior
Huangqi	Astragalus mongholicus/*Astragalus membranaceus*	Fabaceae	-	To boost qi and secure exterior, and draw toxins
Sangbaipi	White Mulberry Root/*Morus alba*	Moraceae	*M. alba*	To disinhibit water and disperse edema
Dangshen	Codonopsis tangshen/*Codonopsis pilosula*	Campanulaceae	*C. pilosula*	To fortify the spleen and supplement lung, boost qi, and engender liquid
Baishao	Common Peony/*Paeonia lactiflora*	Paeoniaceae	*P. lactiflora*	To calm the liver and relieve pain, nourish the blood and constrain yin
Chuanxiong	Cnidium officinale/*Ligusticum chuanxiong*	Apiaceae	*L. chuanxiong*	To move qi, quicken the blood, and dispel wind
Kushen	Light yellow Sophora/*Sophora flavescens*	Fabaceae	*S. flavescens*	To clear heat and dry damp, dispel wind, and kill worms
Juhua	Florists Chrysanthemum Flower/*Chrysanthemum morifolium*	Asteraceae	*C. morifolium*	To dispel wind, clear heat, and resolve toxins
Banxia	Ternate Pinellia/*Pinellia ternata*	Araceae	*P. ternata*	To eliminate damp, resolve cold phlegm, and dissipate binds
Baizhu	Large head Atractylodes/*Atractylodes macrocephala*	Asteraceae	*A. macrocephala*	To boost qi, fortify the spleen, dry damp, and disinhibit water
Fuling	Indian Bread/*Poria cocos*	Fomitopsidaceae	*W. cocos*	To disinhibit water, percolate dampness, fortify the spleen, and quiet the heart
Heshouwu	Tuber Fleeceflower/*Polygonum multiflorum*	Polygonaceae	*F. multiflora*	To enrich yin, nourish the blood, moisten intestines to free stool, dispel wind, and resolve toxins
Danshen	Salvia przewalskii/*Salviae Miltiorrhizae*	Lamiaceae	*S. miltiorrhiza*	To quicken the blood, dispel stasis, nourish the blood, quiet the spirit, cool the blood, and disperse welling abscess
Cheqiancao	All-grass of Rippleseed plantain/*Herba Plantaginis*	Plantaginaceae	*P. asiatica*	Treatment of edema with oliguria, urinary infection with difficult painful urination

TCM = traditional Chinese medicine; UCG = uremic clearance granule.

**Table 2:** Characteristics of Selected Studies.

Study (Year)	Study Design	Inclusion Criteria	No. of Patients	Age (Years)	Route, Dosage, and Frequency	Duration	Inspection Data	Pruritus Severity Assessment	Pruritus Score (Before → After),
Kao et al. 2012 [49]	RCT	HD + HP	U: 16 C: 16	25–74 25–74	Enema, 15 g, 2 times/d	18 M	overall effectiveness	Duo PS	U: 29.18(1.52) → 12.69(3.16) C: 26.54(4.03) → 17.13(3.24)
Yang et al. 2016 [47]	RCT	U: HD + HP (14); HD (7) C: HD + HP (11); HD (10)	U: 21 C: 21	U: 51.48(13.49) C: 51.67(11.68)	Oral, 2.5 g, 2 times/d	1 M	Ca, P, PTH, Scr, BUN, UA, FGF23, CRP, overall effectiveness	Kuypers PS	U: 11.57(2.45) →6.43 (3.02) C: 11.67(4.98) → 11.86(4.33)
Yu et al. 2017 [48]	RCT	HD	U: 65 C: 63	U: 35–68 C: 36–70	Oral, 5 g, 4 times/d	3 M	Ca, P, PTH, β2-MG, hs-CRP, overall effectiveness	VAS	U: 8.17(1.94) → 4.28(1.45) C: 8.21(1.78) → 6.45(1.91)
Sun et al. 2018 [44]	RCT	HD	U: 54 C: 54	U: 54.12(5.78) C: 54.08(6.23)	Oral, 2.5 g, 2 times/d	--	Scr, BUN, UA, hs-CRP, IL-6, TNF-α, Alb, Hb	PS1	U: 11.56(3.02) → 5.12(0.89) C: 11.89(3.12) → 8.28(2.02)
Tan et al. 2018 [45]	RCT	HD + HP	U: 40 C: 40	U: 48.83(8.95) C: 48.92(3.87)	Oral, 5 g, 4 times/d	3 M	Ca, P, PTH, Scr, BUN, UA, IL-6, TNF-α,hs-CRP, overall effectiveness	PS2	U: 8.37(0.89) → 4.6(0.38) C: 8.14(0.96) → 6.44(0.55)
Cao et al. 2019 [40]	RCT	HD	U: 40 C: 40	U: 59.7 C: 59.8	Oral, 5 g, 4 times/d	2 M	hs-CRP, overall effectiveness	NRS	U: 7.89(1.31) → 3.10(0.93) C: 7.95(1.43) → 5.37(1.02)
Kuo et al. 2019 [41]	RCT	HD + Ca+ Calcitriol	U: 30 C: 30	U: 42.6(3.2) C: 41.9(3.4)	Oral, 5 g, 4 times/d	3 M	Ca, P, PTH, overall effectiveness	--	--
Kun et al. 2019 [42]	RCT	HD	U: 23 C: 23	U:45.3(5.3) C:46.1(4.9)	Oral, 5 g, 4 times/d	3 M	overall effectiveness	VAS	U: 8.13(1.77) → 4.73(1.41) C: 8.40(2.07) → 6.28(2.19)
Li et al. 2019 [43]	RCT	HD	U: 52 C: 50	U: 47.2(3.7) C: 46.7(4.2)	Oral, 5 g, 4 times/d	3 M	Ca, P, PTH, β2-MG, hs-CRP, overall effectiveness	VAS	U: 8.18(1.69) → 4.31(1.52) C: 8.20(1.96) → 6.38(1.88)
Chen et al. 2020 [24]	RCT	HD	U: 50 C: 50	U:65.72(10.33) C:64.12(10.54)	Oral, 5 g, 4 times/d	3 M	Ca, P, PTH, SCr, BUN, β2-MG, overall effectiveness, ADR	VAS	U: 7.62(1.02) → 3.36(1.06) C: 7.54(0.98) → 5.53 (1.78)
								5-DIS	U: 17.37(3.56) → 6.44(1.59) C: 16.98(3.72) → 10.82(2.31)
								DLQI	U: 21.84(5.53) → 8.36(2.21) C: 21.54(5.70) → 10.55(3.88)
Xi 2020 [46]	RCT	HD	U: 58 C: 58	U: 47.88(3.52) C: 47.79(3.41)	Oral, 5 g, 4 times/d	--	P, PTH, β2-MG, hs-CRP, overall effectiveness	VAS	U: 7.21(1.72) → 1.47(0.34) C: 7.23(1.71) → 3.48(0.53)

5-DIS = 5-dimensional itching scale; ADR = adverse drug reaction; Alb = albumin; BUN = blood urea nitrogen; C = control group; Ca = calcium; d = day; DLQI = dermatology life quality index; FGF23 = fibroblast growth factor 23; Hb = hemoglobin; HD = hemodialysis; HP = hemoperfusion; hs-CRP = high-sensitivity C-reactive protein; IL-6 = interleukin-6; β2-MG = β2-microglobulin; M = month; NRS = numeric rating scale; P = phosphorus; PS = pruritus scale; PTH = parathyroid hormone; RCT = randomized controlled trial; Scr = serum creatinine; TNF-α = tumor necrosis factor-α; U = uremic clearance granule group; UA = uric acid; UCG = uremic clearance granule group; VAS = visual analog scale.

## Data Availability

The data used to support the findings of this study are included within the article or Supplementary Materials.

## Supplementary Materials

The following are available online at https://www.mdpi.com/article/10.3390/toxins13100702/s1, Table S1: Characteristics of Selected Studies with inspection data, Table S2: Grade profile summary of ‘UCG for UP’ quality assessment, Table S3: Search History.
