# Supplementary material for: Effects of Uremic Clearance Granules in Uremic Pruritus: A Meta-Analysis
Source: Toxins (Basel). 2021 Oct 4;13(10):702. doi: 10.3390/toxins13100702 (PMC8540647; doi:10.3390/toxins13100702)
Supplement: Supplementary file 1 [file toxins-13-00702-s001.zip › toxins-1391143 Supplementary - update.pdf]

Supplementary Materials: Effects of Uremic Clearance Granules in Uremic Pruritus: A Meta-Analysis

Ping-Hsun Lu, Jen-Yu Wang, Hui-En Chuo and Po-Hsuan Lu

Table S1. Characteristics of Selected Studies with inspection data.

Study (year)	Ca	P	PTH	Scr	BUN	UA	TNF-α	IL-6	FGF23	B2-MG	hs-CRP	Alb	Hb	Overall EffectiveNess	ADR
Kao et al. 2011 [55]	--	--	--	--	--	--	--	--	--	--	--	--	--	U: 14/16<br>C:11/16	--
Yang et al. 2016 [53]	U: 2.09(0.24) → 2.30(0.21)<br>C: 2.09(0.25) → 2.10 (0.24)	U: 2.31(0.48) → 1.85(0.41)<br>C: 2.17(0.69) → 2.23(0.66)	U: 562.11(74.42) → 462.46(72.48)<br>C: 574.22(73.45) → 545.25(77.90)	U: 928.25(159.37) → 750.25(143.25)<br>C: 930.33(165.26) → 915.97(146.60)	U: 26.26(4.47) → 19.47(4.22)<br>C: 26.34(4.67) → 24.85(4.13)	U: 527.01 (15.7) → 510.73 (15.32)<br>C: 532.23 (16.9) → 534.18 (15.56)	--	--	U: 777.76(120.88) → 681.38(115.78)<br>C: 779.14(127.78) → 797.33(138.36)	--	U: 13.95(1.02) → 12.02(0.92)<br>C: 14.05(1.11) → 14.22(1.31)	--	--	U: 11/21<br>C: -	--
Yu et al. 2017 [54]	U: 2.29(0.18) →	U: 2.34(0.52) →	U: 432.35(79.16) →	--	--	--	--	--	--	U: 48.57(12.21)→	U: 12.73(3.19) →	--	--	U: 49/65<br>C: 36/63	--

	2.25(0.21)<br>C:<br>2.32(0.17) →<br>2.27(0.20)	1.62(0.36)<br>C:<br>2.28(0.45) →<br>1.94(0.39)	228.83(57.42)<br>C:<br>428.39(74.7) →<br>347.36(67.92)							22.75(8.91)<br>C:<br>50.49(13.46)→<br>34.17(9.06)	6.27(3.96)<br>C:<br>13.09(3.26)→<br>8.75(3.41)				
Sun et al. 2018 [50]	--	--	--	U:<br>356.12(25.45) →<br>212.54(28.78)<br>C:<br>352.89(26.58) →<br>289.78(32.56)	U:<br>20.45(3.56) →<br>10.89(3.02)<br>C:<br>20.78(3.69) →<br>15.42(2.89)	U:<br>128.45(20.12) →<br>154.45(30.12)<br>C:<br>127.89(23.12) →<br>123.78(20.78)	U:<br>115.45(21.45) →<br>75.12(10.56)<br>C:<br>115.02(22.02) →<br>92.45(20.15)	U:<br>23.89(5.02) →<br>12.56(3.02)<br>C:<br>23.78(4.25) →<br>18.57(4.02)	--	--	U:<br>15.78(3.12) →<br>8.79(2.12)<br>C:<br>15.23(3.89) →<br>12.45(3.61)	U:<br>36.45(5.78) →<br>45.12(3.56)<br>C:<br>36.28(5.45) →<br>40.12(5.12)	U:<br>56.89(10.23) →<br>77.454(9.78)<br>C:<br>65.12(6.89) → ()	--	--
Tan et al. 2018 [51]	U:<br>2.27(0.19) →<br>2.34(0.18)<br>C:	U:<br>2.35(0.47) →<br>1.61(0.24)<br>C:	U:<br>486.39(58.21) →<br>271.92(36,04)<br>C:	U:<br>510.47(148.52) →<br>365.23(90.37)<br>C:	U:<br>24.68(5.37) →<br>15.04(2.82)<br>C:	U:<br>381.54(108.52) →<br>247.29(85.47	U:<br>87.34(23.51) →<br>63.4	U:<br>65.82(13.56) →<br>52.65(10.04)	--	--	U:<br>7.13(1.21) →<br>1.45(0.39)<br>C:<br>7.09(1.27)	--	--	U: 38/40<br>C: 31/40	--

	2.29(0.16) → 2.30(0.21)	2.29(0.45) → 1.94(0.32)	489.04(56.83) → 346.38(42.52)	508.91(150.13) → 437.50(99.82)	24.82(5.25) → 20.91(3.26)	) C: 379.05 (109.73) → 314.92 (92.38)	1(13.92) C: 87.46(23.37) → 75.82(18.24)	C: 65.73(13.65) → 60.27(11.19)			→ 3.04(0.73)				
Cao et al. 2019 [46]	--	--	--	--	--	--	--	--	--	--	U: 3.16(1.15) → 2.72(0.81) C: 3.15(1.12) → 3.17(1.06)	--	--	U: 38/40 C:32/40	--
Kuo et al. 2019 [47]	U: 1.75(0.26) → 2.46(0.34) C: 1.72(0.25) → 2.14(0.28)	U: 1.92(0.27) → 1.52(0.16) C: 1.91(0.24) → 1.74(0.18)	U: 241.16(34.48) → 203.36(31.18) C: 240.18(34.22) → 224.65(32.27)	--	--	--	--	--	--	--	--	--	--	U:27/30 C: 19/30	--
Kun et al. 2019 [48]	--	--	--	--	--	--	--	--	--	--	--	--	--	U: 21/23 C: 16/23	

Li et al. 2019 [49]	U: 2.32(0.20) → 2.17(0.18)<br>C: 2.30(0.43) → 2.28(0.17)	U: 2.41(0.49) → 1.59(0.40)<br>C: 2.37(0.43) → 1.96(0.41)	U: 432.41(79.20) → 228.79(57.38)<br>C: 428.42(74.66) → 347.42(67.88)	--	--	--	--	--	--	U: 52.67(12.18) → 22.69(8.87)<br>C: 50.52(13.51) → 34.68(9.37)	U: 12.69(3.21) → 6.31(4.02)<br>C: 13.12(3.31) → 8.81(3.38)			U: 39/52<br>C: 28/50	
Chen et al. 2020 [27]	U: 2.89(0.45) → 1.46(0.17)<br>C: 2.76(0.52) → 2.03(0.19)	U: 2.35(0.34) → 1.82(0.16)<br>C: 2.29(0.30) → 2.05(0.24)	U: 425.54(105.33) → 125.54(78.84)<br>C: 418.87(102.26) → 235.57(86.62)	U: 753.58(40.74) → 284.46(25.57)<br>C: 750.62(42.21) → 487.73(36.22)	U: 28.41(4.33) → 13.44(2.39)<br>C: 27.96(4.82) → 18.87(3.02)	--	--	--	--	U: 14.01(0.97)→ 6.84(0.69)<br>C: 13.87(0.82)→ 11.54(1.06)	--	--	--	U: 42/50<br>C: 33/50	U: 6/50<br>C: 4/50
Xi 2021 [52]	--	U:2.38 (1.41) → 1.42(0.32)<br>C:2.37 (0.42) → 1.94(0.41)	U:430.8(79.3)→ 224.3(56.7)<br>C:431.1(79.2)→ 348.7(67.8)	--	--	--	--	--	--	U:52.57(12.79)→ 21.55(8.24)<br>C:52.68(12.81)→ 34.74(9.38)	U:12.98(3.14)→ 6.29(1.08)<br>C:12.97(3.12)→ 8.74(2.16)	--	--	U:56/58<br>C:45/58	--

ADR = adverse drug reaction; Alb = albumin; BUN = blood urea nitrogen; C = control; Ca = calcium; FGF23 = fibroblast growth factor 23; Hb = hemoglobin; hs-CRP = high-sensitivity C-reactive protein; IL-6 = interleukin-6; iPTH = intact parathyroid hormone; P = phosphorus; Scr = serum creatinine; TNF-α = tumor necrosis factor-α; U = uremic clearance granule; UA = uric acid; β2-MG =β2-microglobulin

Table S2. Grade profile summary of 'UCG for UP' quality assessment.

Primary outcomes

Certainty assessment							№ of patients		Effect		Certainty	Importance
№ of studies	Study design	Risk of bias	Inconsistency	Indirectness	Imprecision	Other considerations	UCG	placebo	Relative (95% CI)	Absolute (95% CI)		
**Visual analog scale**												
5	randomised trials	serious	not serious	not serious	not serious	publication bias strongly suspected	248	244	-	MD **2.02 lower** (2.17 lower to 1.88 lower)	⊕⊕◯◯ LOW	IMPORTANT
**Overall effectiveness**												
9	randomised trials	serious	not serious	not serious	not serious	publication bias strongly suspected	324/374 (86.6%)	251/370 (67.8%)	**OR 3.16** (2.14 to 4.67)	**191 more per 1,000** (from 140 more to 229 more)	⊕⊕◯◯ LOW	IMPORTANT

Secondary outcomes

Certainty assessment							№ of patients		Effect		Certainty	Importance
**№ of studies**	**Study design**	**Risk of bias**	**Inconsistency**	**Indirectness**	**Imprecision**	**Other considerations**	**UCG**	**placebo**	**Relative (95% CI)**	**Absolute (95% CI)**		
**Calcium**												
6	randomised trials	not serious	very serious	not serious	serious	publication bias strongly suspected	258	254	-	MD **0.08 higher** (0.26 lower to 0.43 higher)	⨁◯◯◯ VERY LOW	IMPORTANT
**Phosphorus**												
7	randomised trials	not serious	serious	not serious	not serious	publication bias strongly suspected	316	312	-	MD **0.32 lower** (0.41 lower to 0.24 lower)	⨁⨁◯◯ LOW	IMPORTANT

PTH

Certainty assessment							№ of patients		Effect		Certainty	Importance
**№ of studies**	**Study design**	**Risk of bias**	**Inconsistency**	**Indirectness**	**Imprecision**	**Other considerations**	**UCG**	**placebo**	**Relative (95% CI)**	**Absolute (95% CI)**		
5	randomised trials	not serious	very serious	not serious	not serious	publication bias strongly suspected	226	222	-	MD **93.03 lower** (141.91 lower to 44.14 lower)	⊕◯◯◯ VERY LOW	IMPORTANT
**iPTH**												
2	randomised trials	not serious	serious	not serious	not serious	publication bias strongly suspected	90	90	-	MD **89.51 lower** (123.95 lower to 55.07 lower)	⊕⊕◯◯ LOW	IMPORTANT
**hs-CRP**												
6	randomised trials	not serious	very serious	not serious	not serious	publication bias strongly suspected	309	305	-	MD **2.07 lower** (2.89 lower to 1.25 lower)	⊕◯◯◯ VERY LOW	IMPORTANT

**TNF-α**												
2	randomised trials	not serious	not serious	not serious	not serious	publication bias strongly suspected	94	94	-	MD **15.23 lower** (20 lower to 10.47 lower)	⊕⊕⊕◯ MODERATE	IMPORTANT
**IL-6**												
2	randomised trials	not serious	not serious	not serious	not serious	publication bias strongly suspected	94	94	-	MD **6.13 lower** (7.42 lower to 4.84 lower)	⊕⊕⊕◯ MODERATE	IMPORTANT
**SCr**												
4	randomised trials	not serious	very serious	not serious	not serious	publication bias strongly suspected	165	165	-	MD **128.38 lower** (214.34 lower to 42.42 lower)	⊕◯◯◯ VERY LOW	IMPORTANT
**BUN**												
4	randomised trials	not serious	not serious	not serious	not serious	publication bias strongly suspected	165	165	-	MD **5.23 lower** (5.87 lower to 4.58 lower)	⊕⊕⊕◯ MODERATE	IMPORTANT

UA

3	randomised trials	not serious	very serious	not serious	serious	publication bias strongly suspected	115	115	-	MD **16.94 lower** (63.56 lower to 29.67 higher)	⊕◯◯◯ VERY LOW	IMPORTANT

BUN: blood urea nitrogen; CI: Confidence interval; hs-CRP: high sensitivity C- reactive protein; IL-6: interleukin 6; iPTH: parathyroid hormone intact; MD: Mean difference; OR: Odds ratio; PTH: parathyroid hormone; Scr: serum creatinine; TNF-α: tumor necrosis factor-alpha; UA: uric acid; β2-MG: beta 2-Microglobulin

Table S3. Search_History.

Pubmed

No.	Query	Results	Date
#6	#3 AND #4 AND #5	68	19-Jul-21
#5	'uremic clearance' OR (uremic AND ('clearance'/exp OR clearance)) OR 'uremic clearance granule' OR (uremic AND ('clearance'/exp OR clearance) AND ('granule'/exp OR granule)) OR niaoduqing OR 'niaoduqing particles' OR (niaoduqing AND particles) OR 'niaoduqing keli' OR (niaoduqing AND keli)	1842	19-Jul-21
#4	'pruritus'/exp OR pruritus OR 'pruritis'/exp OR pruritis OR itch* OR 'xerosis'/exp OR xerosis OR 'skin problems' OR (('skin'/exp OR skin) AND problems) OR 'skin disorders' OR (('skin'/exp OR skin) AND ('disorders'/exp OR disorders))	1189856	19-Jul-21
#3	#1 OR #2	875885	19-Jul-21
#2	uremic OR 'uremia'/exp OR uremia OR uremias	56328	19-Jul-21
#1	'chronic kidney disease'/exp OR 'chronic kidney disease' OR (chronic AND ('kidney'/exp OR kidney) AND ('disease'/exp OR disease)) OR 'kidney injury'/exp OR 'kidney injury' OR (('kidney'/exp OR kidney) AND ('injury'/exp OR injury)) OR 'kidney failure'/exp OR 'kidney failure' OR (('kidney'/exp OR kidney) AND ('failure'/exp OR failure)) OR 'chronic renal failure'/exp OR 'chronic renal failure' OR (chronic AND ('renal'/exp OR renal) AND ('failure'/exp OR failure)) OR 'end-stage renal disease'/exp OR 'end-stage renal disease' OR ('end stage' AND ('renal'/exp OR renal) AND ('disease'/exp OR disease)) OR 'end stage renal disease'/exp OR 'end stage renal disease' OR (end AND stage AND ('renal'/exp OR renal) AND ('disease'/exp OR disease)) OR 'dialysis'/exp OR dialysis OR 'hemodialysis'/exp OR hemodialysis OR 'peritoneal dialysis'/exp OR 'peritoneal dialysis' OR (peritoneal AND ('dialysis'/exp OR dialysis))	865941	19-Jul-21

Cochrane Library

ID	Search	Hits

#1	chronic kidney disease OR kidney injury OR kidney failure OR chronic renal failure OR end-stage renal disease OR end stage renal disease OR dialysis OR hemodialysis OR peritoneal dialysis	45967
#2	uremic OR Uremia OR uremias	2081
#3	#1 OR #2	46362
#4	Pruritus OR Pruritis OR itch* OR xerosis OR skin problems OR skin disorders	23466
#5	uremic clearance OR uremic clearance granule OR Niaoduqing OR Niaoduqing Particles OR Niaoduqing KeLi	194
#6	#3 AND #4 AND #5	11
	其中有一篇歸屬於 Special collection 的 "Neglected tropical diseases: the top five"無法匯出至 EndNote	https://www.cochranelibrary.com/collections/doi/10.1002/14651858.SC000021/full

Embase

No.	Query	Results	Date
#6	#3 AND #4 AND #5	68	19-Jul-21
#5	'uremic clearance' OR (uremic AND ('clearance'/exp OR clearance)) OR 'uremic clearance granule' OR (uremic AND ('clearance'/exp OR clearance) AND ('granule'/exp OR granule)) OR niaoduqing OR 'niaoduqing particles' OR (niaoduqing AND particles) OR 'niaoduqing keli' OR (niaoduqing AND keli)	1842	19-Jul-21
#4	'pruritus'/exp OR pruritus OR 'pruritis'/exp OR pruritis OR itch* OR 'xerosis'/exp OR xerosis OR 'skin problems' OR (('skin'/exp OR skin) AND problems) OR 'skin disorders' OR (('skin'/exp OR skin) AND ('disorders'/exp OR disorders))	1189856	19-Jul-21
#3	#1 OR #2	875885	19-Jul-21

#2	uremic OR 'uremia'/exp OR uremia OR uremias	56328	19-Jul-21
#1	'chronic kidney disease'/exp OR 'chronic kidney disease' OR (chronic AND ('kidney'/exp OR kidney) AND ('disease'/exp OR disease)) OR 'kidney injury'/exp OR 'kidney injury' OR (('kidney'/exp OR kidney) AND ('injury'/exp OR injury)) OR 'kidney failure'/exp OR 'kidney failure' OR (('kidney'/exp OR kidney) AND ('failure'/exp OR failure)) OR 'chronic renal failure'/exp OR 'chronic renal failure' OR (chronic AND ('renal'/exp OR renal) AND ('failure'/exp OR failure)) OR 'end-stage renal disease'/exp OR 'end-stage renal disease' OR ('end stage' AND ('renal'/exp OR renal) AND ('disease'/exp OR disease)) OR 'end stage renal disease'/exp OR 'end stage renal disease' OR (end AND stage AND ('renal'/exp OR renal) AND ('disease'/exp OR disease)) OR 'dialysis'/exp OR dialysis OR 'hemodialysis'/exp OR hemodialysis OR 'peritoneal dialysis'/exp OR 'peritoneal dialysis' OR (peritoneal AND ('dialysis'/exp OR dialysis))	865941	19-Jul-21

CINAHL

#	查詢	設限值/擴增詞	上次執行透過	結果
S6	S3 AND S4 AND S5	擴增詞 - 套用相等主題	介面 - EBSCOhost Research Databases	2
		檢索模式 - 尋找全部檢索詞語	檢索畫面 - 進階檢索	
			資料庫 - CINAHL	
S5	uremic clearance OR uremic clearance granule OR Niaoduqing OR Niaoduqing Particles OR Niaoduqing KeLi	擴增詞 - 套用相等主題	介面 - EBSCOhost Research Databases	94
		檢索模式 - 尋找全部檢索詞語	檢索畫面 - 進階檢索	
			資料庫 - CINAHL	
S4	Pruritus OR Pruritis OR itch* OR xerosis OR skin problems OR skin disorders	擴增詞 - 套用相等主題	介面 - EBSCOhost Research Databases	27,313
		檢索模式 - 尋找全部檢索詞語	檢索畫面 - 進階檢索	
			資料庫 - CINAHL	
S3	S1 OR S2	擴增詞 - 套用相等主題	介面 - EBSCOhost Research Databases	91,429
		檢索模式 - 尋找全部檢索詞語	檢索畫面 - 進階檢索	
			資料庫 - CINAHL	
S2	uremic OR Uremia OR uremias	擴增詞 - 套用相等主題	介面 - EBSCOhost Research Databases	4,043
		檢索模式 - 尋找全部檢索詞語	檢索畫面 - 進階檢索	
			資料庫 - CINAHL	
S1	chronic kidney disease OR kidney injury OR kidney failure OR chronic renal failure OR end-stage renal disease OR	擴增詞 - 套用相等主題	介面 - EBSCOhost Research Databases	89,747
		檢索模式 - 尋找全部檢索詞語	檢索畫面 - 進階檢索	

	end stage renal disease OR dialysis OR hemodialysis OR peritoneal dialysis		資料庫 - CINAHL	

ClinicalTrials.gov

Condition or disease	uremic OR Uremia OR Uremias OR Atypical Hemolytic Uremic Syndrome OR pruritus
**Other terms**	uremic clearance OR Niaoduqing OR Niaoduqing KeLi
搜尋結果為 17 篇	

Airiti Library

chronic kidney disease OR kidney failure OR dialysis OR 透析 OR 血透 OR 腹透 OR 腎衰竭 OR 腎功能衰竭 OR 腎功能不全 OR 腎小球腎炎 OR 腎炎 OR uremic OR Uremia OR uremias OR 尿毒	篇名.關鍵字.摘要
pruritus OR Pruritis OR itch* OR xerosis OR Skin Diseases OR 皮膚搔癢 OR 搔癢 OR 癢	篇名.關鍵字.摘要
uremic clearance OR Niaoduqing OR Niaoduqing KeLi OR 尿毒清颗粒 OR 尿毒清	篇名.關鍵字.摘要
三項以 AND 串連共搜尋到 8 篇文獻	
搜尋指令為以下	
((chronic kidney disease OR kidney failure OR dialysis OR 透析 OR 血透 OR 腹透 OR 腎衰竭 OR 腎功能衰竭 OR 腎功能不全 OR 腎小球腎炎 OR 腎炎 OR uremic OR Uremia OR uremias OR 尿毒)) = 篇名.關鍵字.摘要 AND ((pruritus OR Pruritis OR itch* OR xerosis OR Skin Diseases OR 皮膚搔癢 OR 搔癢 OR 癢)) = 篇名.關鍵字.摘要 AND ((uremic clearance OR Niaoduqing OR Niaoduqing KeLi OR 尿毒清颗粒 OR 尿毒清)) = 篇名.關鍵字.摘要	

Wanfang databases

chronic kidney disease OR kidney injury OR kidney failure OR chronic renal failure OR chronic kidney disease OR end-stage renal disease OR dialysis OR hemodialysis OR peritoneal dialysis OR 透析 OR 血透 OR 腹透 OR 腎衰竭 OR 腎功能衰竭 OR 腎功能不全 OR 腎小球腎炎 OR 腎炎 OR uremic OR Uremia OR uremias OR 尿毒	全部
pruritus OR pruritus OR Pruritis OR itch OR xerosis OR skin problems OR skin disorders OR 皮膚搔癢 OR 搔癢 OR 癢	全部
uremic clearance OR uremic clearance granule OR Niaoduqing OR Niaoduqing Particles OR Niaoduqing KeLi OR 尿毒清颗粒 OR 尿毒清	全部
三項以 AND 串連共搜尋到 82 篇文獻	

Chinese National Knowledge Infrastructure

#1	尿毒清	篇關摘
#2	皮肤瘙痒	篇關摘
	二項以 AND 串連共搜尋到 18 篇文獻	
